# Targeting doxorubicin encapsulated in stealth liposomes to solid tumors by non thermal diode laser

**DOI:** 10.1186/s12944-016-0235-2

**Published:** 2016-04-05

**Authors:** Magdy M. Ghannam, Reem El Gebaly, Maha Fadel

**Affiliations:** Department of Physics and Astronomy, King Saud University, Riyadh, Saudi Arabia; Biophysics Department, Faculty of Science, Cairo University, Giza, Egypt; Department of Applied Laser Tech, National Institute of Laser Sc, Cairo University, Giza, Egypt

**Keywords:** Liposomes, Doxorubicin, Diode laser, Anticancer

## Abstract

**Background:**

The use of liposomes as drug delivery systems is the most promising technique for targeting drug especially for anticancer therapy.

**Methods:**

In this study sterically stabilized liposomes was prepared from DPPC/Cholesterol/PEG-PE encapsulated doxorubicin. The effect of lyophilization on liposomal stability and hence expiration date were studied. Moreover, the effect of diode laser on the drug released from liposomesin vitro and in vivo in mice carrying implanted solid tumor were also studied.

**Results:**

The results indicated that lyophilization of the prepared liposomes encapsulating doxorubicin led to marked stability when stored at 5 °C and it is possible to use the re-hydrated lyophilized liposomes within 12 days post reconstitution. Moreover, the use of low energy diode laser for targeting anticancer drug to the tumor cells is a promising method in cancer therapy.

**Conclusion:**

We can conclude that lyophilization of the liposomes encapsulating doxorubicin lead to marked stability for the liposomes when stored at 5°C. Moreover, the use of low energy diode laser for targeting anticancer drug to the tumor cells through the use of photosensitive sterically stabilized liposomes loaded with doxorubicin is a promising method. It proved to be applicable and successful for treatment of Ehrlich solid tumors implanted in mice and eliminated toxic side effects of doxorubicin.

## Background

Targeting drugs through carrier system have been a promising theme in therapeutics research. It is usually attained by utilizing a carrier e.g., albumin conjugates, antibodies, lectine, glycoproteins, DNA, dextran, polysaccharides, nanoparticles and liposomes [1–6]. There is widespread interest of using liposomes as drug carriers, which requires pharmaceutically acceptable procedures for scaling up to larger batch sizes, stable and economically feasible.

Liposomes can be defined as vesicles in which an aqueous phase is entirely enclosed by one or several membranes composed of phospholipid molecules. It can be constructed to entrap quantities of materials within their aqueous compartment and/or within the membranes [7–12]. Liposomes have been widely studied in medically-related fields as capsules for in vivo delivery of therapeutic agents [5, 13–15]. Since liposomes are used as carrier vehicles for anticancer drugs [6, 16], it is better to target the drug to the site of action, enhance and sustain its clinical effects, reduce its toxicity and protect it from metabolism and immune responses [15, 17, 18].

There are four main strategies to target drugs encapsulated liposomes. These strategies include antibody - coated liposomes which are specifically bound to antigen - presenting target cells. These liposomes facilitate the uptake by macrophage [19]. Thermosensitive liposomes, which are undergoing phase transition at a specific temperature, are able to release their content at a desirable temperature since the permeability of liposomal membrane increases drastically at its phase transition [20]. When the target is warmed, liposomes release their drug content as they pass through the site [21]. The third strategy is the use of pH-sensitive liposomes where their membranes are stabilized by the addition of a specific materials, such as fatty acids, which are charged at neutral pH but lose their charge at low pH destabilizing the vesicles [22]. Sensitive pH liposomes can fuse with biomembrane and/or destabilize at low pH. The fourth strategy depends on the fact that liposomes can be stabilized by the presence of proteins, typically by anchoring a specific antibody in the membrane through covalent attachment of fatty acid chains or other lipid molecules. When these legends aggregate by binding to the target, their ability to stabilize the membrane is reduced and liposomes disintegrate releasing their content [23–25].

Doxorubicin (DOX) is one of the most valuable anticancer drugs in the present clinical use. The use of DOX within liposomes markedly reduces its cardio toxicity without loss of its anticancer activity [15].

The aim of this study is to investigate the effect of non-thermal laser dose on the drug release mechanism from liposomes in-vitro and in- vivo studies.

## Methods

### Materials

1,2 dipalmitoyl-sn-phosphatidylecholine (DPPC) of molecular weight 743, Cholesterol (CHOL) of molecular weight 386.7, Lactose monohydrate and HEPS buffer, N-(2-Hydroxyl) peperazine-N-(2-ethanosulphonic acid), were purchased from Sigma Chemicals St. Louis, USA. Distearyl-phosphatidyl ethanolamine derivative at the amino position with a molecular weight 2000 segment of polyethylene glycol (PEG-PE) was obtained from Liposome Technology (LTI) USA. Doxorubicin hydrochloride (DOX) was purchased in 10 mg - vial as freeze dried powder from Farmitalia Research Laboratories, Milano, Italy. Ammonium sulfate of purity 98 % was obtained from El Nasr Chemical Company, Egypt. Sephadex G-75 superfine purchased from Pharmacia Fine Chemicals, with beads particle size 25–100 μm (The gel column was of 2.0 cm in diameter and 30 cm in height). All chemicals were used without further purification.

### Methods

#### Liposomes preparation and drug encapsulation

In this work, sterically stabilized liposomes (stealth) were prepared from DPPC/Chol/PEG-PE at the molar ratios 100:20:4, respectively. The lipids were dissolved in chloroform and then deposited from organic solvent in a thin film on the walls of the round bottom flask of the rotary evaporator under reduced pressure and nitrogen gas. Ammonium sulfate (250 mM at pH 4) was then added to hydrate the dried thin film in the flask and kept in the water bath at 55 °C for hydration. To get small vesicles, the suspension is sonicated under temperature control for a period of 2 h. The sample was then gently poured on a surface of the gel chromatographic column packed with sephadex G-75 for the removal of un-entrapped ammonium sulfate.

Doxorubicin Hydrochloride (DOX) of 10 mg was dissolved in 5 ml HEPS buffer at pH 7.4and then added to the liposome suspension that eluted from the gel column at a concentration of 1 mg DOX/10 mol. of phospholipid. The liposomes - DOX mixture was incubated in water bath of a rotary evaporator for 1 h at 55 °C under reduced pressure. Post incubation period, the sample was passed again in the gel column to remove non-encapsulated DOX. The drug loading took place by the pH gradient method [26].

#### Liposomes lyophilization and characterization

The lyophilization process was taken through the use of a freeze-drying system type Lyph-lock® 4.5 l manufactured by Labconco Corporation USA*.* The prepared liposomes encapsulating Dox were centrifuged for 15 min at 10,000 rpm and temperature of 15 °C. Liposomes pellet was then added to 10 % lactose monohydrate solution which protects liposomes against fusion and leakage during lyophilization process [27, 28]. Liposomes suspension was introduced into 2 ml- vials at a concentration 83 μg/ml. The vials were then freeze at −70 °C before being attached to the freeze drying system. The samples in the vials were then left for 24 h in the freeze dryer till a dry cake was formed. Characterizations of the lyophilized liposomes were carried out by measuring the following parameters:

##### Size distribution

Freshly prepared slides of the loaded liposomes were scanned through the use of image analyzer type SMAICA Systems with Ziess AXIOTRON microscope (ELBEK GmbH, Germany). In this system, the liposomes slides were imaged microscopically through the use of an electronic camera which generates an electronic signal proportional to the intensity of illumination. Consequently, the full measurements of the size and size distribution of the examined liposomes can be recorded.

##### Drug release

The drug release from the lyophilized liposomes in buffer was studied.

Two milliliter of HEPS buffer was added to a vial containing lyophilized liposomes. The vials were centrifuged at 10,000 rpm for 15 min at −20 °C. So the supernatant was sucked out.

The drug absorbance (A)in the supernatant was measured using a spectrophotometer (Shimatzu 1601PC, Japan) at the characteristic absorption band of doxorubicin (500 nm). So, the drug concentration, [Drug] was calculated using an experimental standard calibration curve which is represented by the equation:

1 $$ \left[\mathrm{Drug}\right]=58.5\mathrm{A}{\textstyle \hbox{-} }0.82 $$

The sample was then incubated at 37 °C and the amount of drug released was calculated after different incubation periods up to 24 h.

##### Liposomes stability

Lyophilized and unlyophilized liposomes were stored on at 5 °C for periods up to 12 months. The following examinations were carried out on the shelf stored liposomes every 2 months:

*Size*: The liposomes size was periodically examined through the use of the image analyzer and its average size was estimated.

*Release and expiratory date*: The concentration of the encapsulated drug was measured as a function of the storage time. So the expiratory date of the constructed liposomes shelf-life (T_**90**_) for lyophilized and unlyophilized liposomes was calculated.

#### Effect of laser on drug release

Diode laser of 250 mW and 650 nm continuous wave (CW), Type DC BRUSHLESS PAT.PENDING Corp., Japan, was used. The deposited energy in Joules from the CW laser was calculated from the equation:

$$ \mathrm{Energy}\left(\mathrm{J}\right)=\mathrm{Laser}\mathrm{power}\left(\mathrm{J}/\mathrm{s}\right)\ast \mathrm{exposure}\mathrm{time}\left(\mathrm{s}\right) $$

##### In-vitro studies

The drug release rate from lyophilized liposomes was studied before and after exposure to different laser energies deposited in the sample. To follow the release, 2 ml of saline were added to a vial containing lyophilized liposomes. The sample was then exposed to laser for different exposure periods (i.e., different deposited energies). It is worthy to say that there was no measurable temperature increase of the exposed liposomes during laser irradiation. The absorbance of the sample was then measured as a function of irradiation dose at 37 °C, considering the value of the absorbance of the sample before irradiation with laser as reference (i.e., zero level).

##### In-vivo studies

Forty male BALB/C mice with average weight 18 ± 2.0 g were used. The mice were inoculated subcutaneously into the left flank with 1 × 10^6^ single cell suspension isolated from Ehrlich ascites carcinomas. Animals injected with tumor cells were classified into four groups namely A, B, C and D, each was of 10 animals. They undertake for the following protocol:

- ***Group A***: control group which didn’t receive any drug or external treatment.
- ***Group B***: received a single dose of free doxorubicin (DOX) of 2 μg/g of animal body weight (~40 μg DOX) injected intraperitoneally at the7^th^day post tumor implantation.
- ***Group C***: received a single dose of about 40 μg doxorubicin encapsulated sterically stabilized liposomes suspension injected intraperitoneally at the 7th day post tumor implantation in the animal.
- ***Group D***: received the same amount of drug encapsulated liposomes as groups C injected intraperitoneally and treated by CW diode laser (250 mW power and 75 J energy deposited) after one hour post injection.

#### Tumor studies included

##### Tumor growth and survival period

Tumor growth was followed by measuring the three mutually orthogonal tumor diameters with a caliper. The volume of the tumor (V) was calculated from the tumor dimensions, length a, width b and height c, which is given by the following equation [15]:

2 $$ \mathrm{V}=\pi \left(\mathrm{a}\mathrm{b}\mathrm{c}\right)/6 $$

The tumor size was measured three times weekly starting after the 7^th^day post tumor implantation. The day of death of each mouse from each group was recorded and the percentage of surviving animals was calculated. Experiment was terminated 90 days after tumor implantation.

##### Histological examination

Thirty five days after tumor implantation part of the mice from groups A and B was sacrificed and biopsies were performed. Tumors from groups C and D were examined just before animal death with 1 or 2 days. Tumors were excised and pathologically examined using light microscope attached with camera.

### Statistical analysis

The statistical methods and analysis for evaluation of the results were done by calculating arithmetic means and standard deviations for all groups. The Mann-Whitney *U*-test was used to determine the significant differences among values of different groups. A P < 0.05 was considered significant.

### Animal ethics

All animal procedures and care were performed using guidelines for the care and use of laboratory animal ethics committee of King Saud University.

## Results and discussion

### In-vitro studies

#### Characteristics of the lyophilized liposomes

**Fig. 1:**
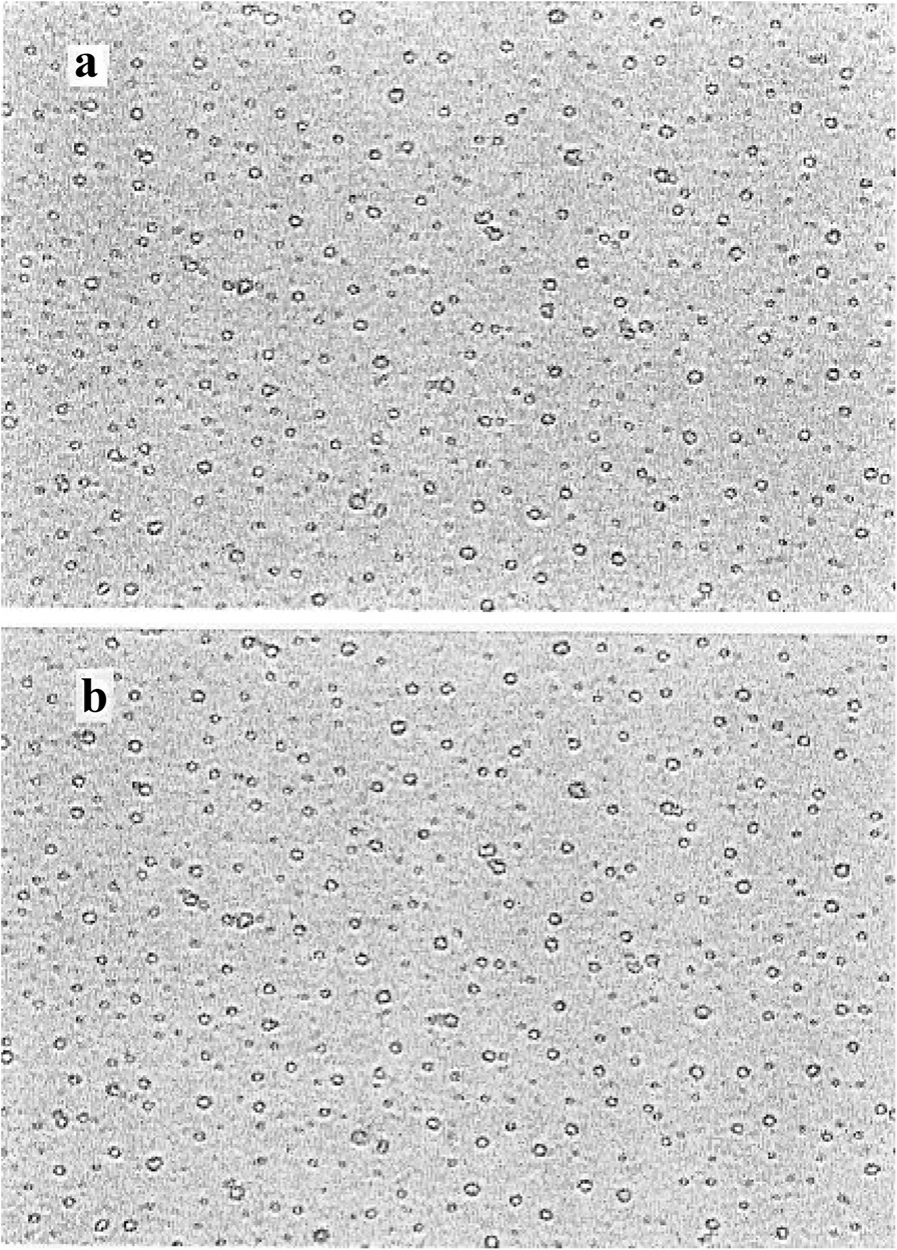
Photographs recorded by the image analyzer system for: **a** Freshly prepared drug loaded liposomes and **b** The same liposomes after being shelf stored at 5 °C for a period of one year

**Fig. 2:**
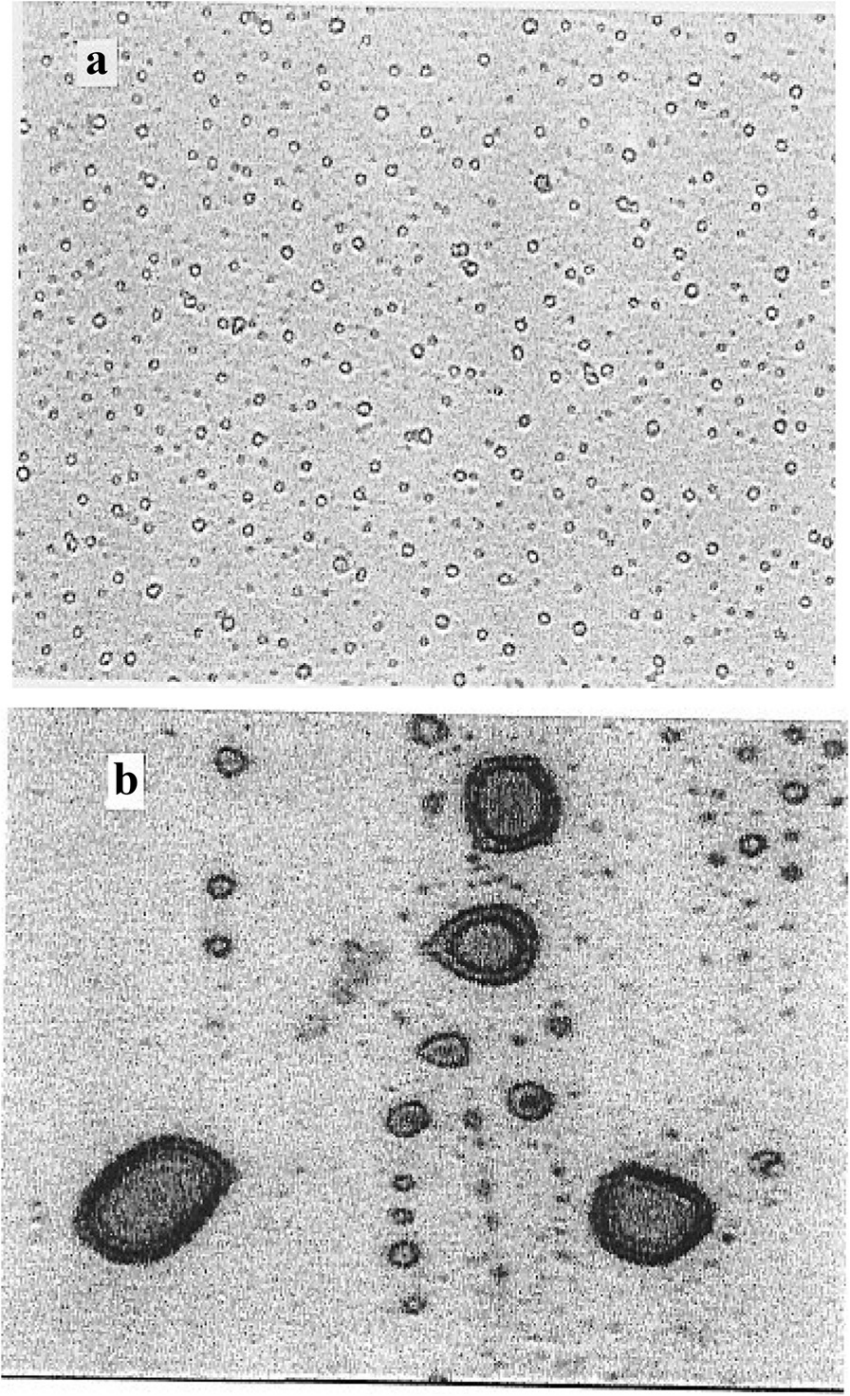
Images for: **a** Rehydrated lyophilized liposomes and **b** The same liposomes after being shelf stored at 5 °C for a period of one year respectively

Figure 1a, b represents photographs recorded by the image analyzer system for the freshly prepared drug loaded liposomes and the same liposomes after being shelf stored at 5 °C for a period of one year respectively. The results indicate that its average diameter is about 9.2 μm. Figure 2a, b shows images for the rehydrated lyophilized liposomes and the same liposomes after being shelf stored at 5 °C for a period of one year. Liposomal size distribution indicated that there is no measurable change in the form and diameter due to lyophilization except the appearance of few clusters. Moreover, shelf storage of the lyophilized liposomes didn’t cause measurable change in their size.

The drug released from the lyophilized and unlyophilized drug loaded sterically stabilized liposomes was measured every 3 months after storage at 5 °C for periods up to 1 year.

**Fig. 3:**
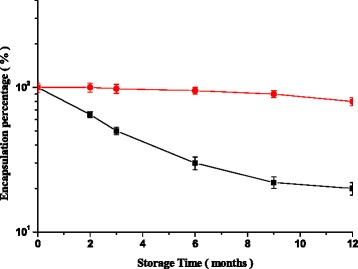
Variation of the encapsulated drug percentage in the lyophilized (red circle) and unlyophilized (black square) liposomes as a function of shelf storage period. * Significant (*p* < 0.05) encapsulation drug percentage compared to unlyophilized

Figure 3 illustrates the variation of the encapsulated drug percentage in the lyophilized and unlyophilized liposomes as a function of shelf storage period. The results indicate that the expiratory date (T_90_) of the constructed lyophilized liposomes is 7.75 months and about 12 days for the unlyophilized liposomes. The values of the released and encapsulated drug in the liposomes after incubation at 37 °C for different periods up to 20 h is demonstrate in Table 1.

**Table 1:** Drug released from liposomes and drug encapsulated after incubation at 37 °C for different periods

Incubation time (hours)	Drug released conc. (μg/ml)	Drug encapsulated conc. (μg/ml)	Percentage drug encapsulated	Percentage drug released
0.0	0.0	69.4	100	0
0.5	2.5 ± 0.02^a^(S)	66.9	96.3	3.7
1.0	3.1 ± 0.02^a^(S)	66.3	95.5	4.5
3.0	3.2 ± 0.04^a^(S)	66.2	95.3	4.7
5.0	4.1 ± 0.03^a^(S)	65.3	94.1	5.91
7.0	5.8 ± 0.05^a^(S)	63.6	91.6	8.4
10.0	7.2 ± 0.05^a^(S)	62.2	89.6	10.4
20.0	7.7 ± 0.02^a^(S)	61.7	88.9	11.1

^a^Compared to control group, *S* Significant when *p* < 0.05, *NS* not significant (*P* > 0.05)

#### Effect of irradiation with continuous wave diode laser

**Fig. 4:**
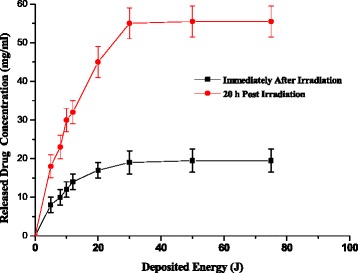
Variation of the drug release concentration as a function of laser energy deposited measured directly after irradiation and 20 h post irradiation. * Significant (*p* < 0.05) drug release concentration measured 20 h post irradiation compared to measured directly after irradiation

Seven samples from the same vial were exposed to different deposited energies from the diode laser. Figure 4 shows the variation of the drug release concentration as a function of laser energy deposited measured directly after irradiation and 20 h post irradiation. The maximum release of the drug occurred at energy of 30 J. Higher energies did not show considerable increase in the amount of released drug. After liposomal exposure to laser energy of 30 J, 33.6 % of the encapsulated drug was released and it reached about 95 % after 20 h post irradiation. This continuous release of drug after stopping irradiation with laser can be explained as follows:

1. The drug loading methods used in the preparation of sterically stabilized liposomes is the ammonium sulfate gradient method. In this method, drug was loaded due to its shuttle into the liposomes forming complex gel of [(DOX)_2_ SO_4_]_n_ in excess ammonium sulfate.
2. These liposomes when irradiated with laser and release amount of their encapsulated drugs, some of the excess ammonium ions encapsulated in the liposomes will be released and cause more leakage from other liposomes [29].
3. This process propagates and enhances the drug released from liposomes as far as ammonia ion concentration in the medium is increased. A process occurs as a feedback mechanism.

It may be presumed that irradiation with non-thermal laser, results in activation of the liposomes, which will result in the release of the drug from the irradiated liposome. The release of excess ammonium sulfate ions from the liposomes will in turn cause further release of drug and ammonium sulfate from other liposomes. Laser energy from the CW diode could work as a powerful initiator for drug release and the ammonia released during the process is responsible for the delayed drug release mechanism.

#### In-vivo studies

**Fig. 5:**
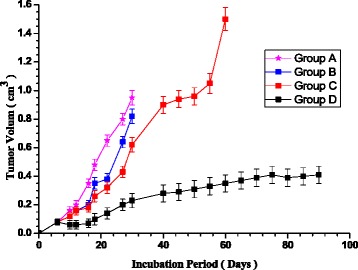
Variation of the average tumor size with the incubation period for all animals from the groups; A (control group), B (received a single dose of free doxorubicin), C (received a single dose doxorubicin encapsulated liposomes), and D (received as groups C injected and treated by CW diode laser). * Significant (*p* < 0.05) increase in tumor size for groups A, B & C. ** Not significant (*P* > 0.05) increase in tumor size for group D from day 30 till the day 90

The tumor size was measured seven days post tumor implantation in the animals. Figure 5 shows the variation of the average tumor size with the incubation period for all animals from the groups A, B, C, and D. Injection of the animals with free doxorubicin slightly inhibited tumor growth (group B) as compared with group A. Animals from groups A and B did not survive more than 30 days post injection with the tumor. The tumor growth in group C was much slower than that of groups A and B. The animals in group C survived till the day 60 post injection with tumor. Therefore, the injection of drug loaded liposomes has the advantage of protecting the drug from metabolism and immune responses which rendered it effective for longer period [17, 18]. Moreover, the toxic side effects of doxorubicin to sensitive organs such as heart, bone marrow and erythrocyte membrane may be reduced [26, 30, 32]. At the day of animal death of group C, the average tumor size was about 1.5 cm^3^ while at day of death of animals of groups A and B the average tumor size was only 0.93 and 0.85 cm^3^ respectively.

For animals of group D, the tumor growth rate was minimized as compared with animals from other groups till day 30 post injection of the tumor in the animals. Insignificant increase of the tumor size occurred from the day 30 till the day 90 when the animals were sacrificed and plateau like dependence of the tumor size on the incubation period is noticed.

**Fig. 6:**
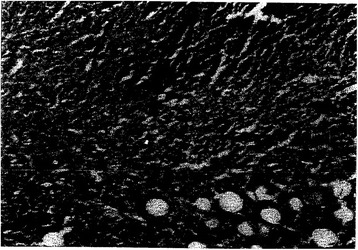
Light microscope photograph for a histological section in a tumor from animals of group A

Histological examination of samples took place at day 28 post tumor implantation for animals of groups A and B. Therefore, the histological examination of the tumors of group C was managed at day 60 and of group D at day 90. Animals from group D were active similar to normal group till day 90 when they were sacrificed. Figure 6 represents a light microscope photograph for a histological section in a tumor from animals of group A. It is clear that the neoplastic cells with hyper chromatic and pleomorphic nuclei and scant eosinophilic cytoplasm are arranged in sheets. These neoplastic cells vary in size and shape. Histological section for group B showed the presence of islands of aggregates of neoplastic cells. These cells vary in size and shape. Islands of normal subcutaneous tissue were seen between the aggregates of tumor cells. Moreover, smaller islands of aggregated neoplastic cells and there are islands of necrotic cells are seen in the histological section of group C.

**Fig. 7:**
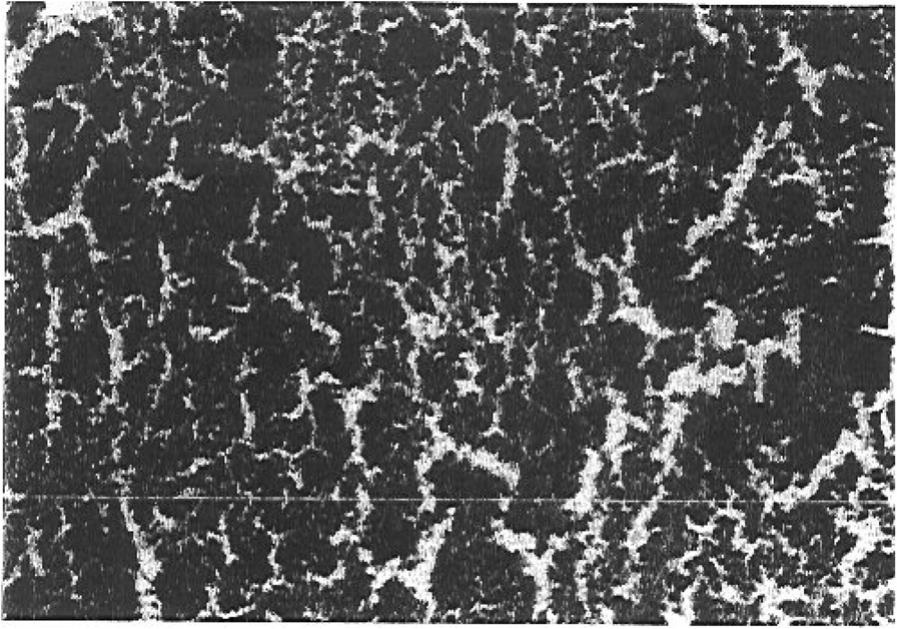
Light microscope photograph for a histological section in tumor of animals of group D

Figure 7 shows a photograph from a light microscope for a histological section in the tumor of animals of group D. It is clear from the figure that there is complete cure of the aggregates of neoplastic cells with normal subcutaneous tissue.

An important site of cytotoxic action of the anticancer drug doxorubicin is the nucleus, where doxorubicin intercalates into DNA, forming DNA adducts and inhibiting topoisomerase II. When free doxorubicin reaches the tumor site, doxorubicin that is released from liposomes within the tumor interstitial space is capable of diffusing widely within the tumor. Doxorubicin can diffuse into surrounding cell membrane or protein associated, or diffuses into subcellular compartments such as mitochondria and nuclei [14, 33].

The histological examination of the tumor from the animals from groups A, B, C, and D proved that animals injection with sterically stabilized liposomes loaded with doxorubicin and targeted to the tumor by non-thermal diode laser energy of 70 J in one shot during 5 min administration gave complete curing of the tumor and no further tumor growth was measured. Sixty percent of the animals survived longer than day 100 post implantation of the tumor. The death of the 40 % of the animals before day 100 may be due to the flow of some tumor cells in the blood stream (during injection process by tumor cells) to other untreated organs with laser which may lead to acceleration of animal death. These findings are supported by a number of researches considering the antitumor effectiveness of electrotherapy or electro-chemotherapy [34–37].

DOX-loaded liposomes have enhanced efficacy in some solid tumors compared with free doxorubicin, because they passively target solid tumors through the enhanced permeability and retention effect [38, 39], resulting in increased drug payloads delivered to tumors. The enhanced permeability and retention effect are a result of defective vascular endothelial linings of growing tumors, resulting in gaps in the endothelium up to f800 nm in diameter, which are large enough to permit the extravasation of liposomes with diameters in the range of 100 nm [40]. In addition, growing tumors have defective lymphatic drainage, which contributes to the extended residence time of extravagated liposomes in the interstitial space of the tumor. Liposomes residing in the interstitial space gradually release their entrapped drug, exerting antitumor effects.

## Conclusion

We can conclude that lyophilization of the liposomes encapsulating doxorubicin lead to marked stability for the liposomes when stored at 5 °C. It is possible to use rehydrated lyophilized liposomes within 12 h post rehydration, in case of being stored at 5 °C. The size of the tumor is not the only marker for the survival period of the animal. The use of low energy diode laser for targeting anticancer drug to the tumor cells through the use of photosensitive sterically stabilized liposomes from DPPC-Chol-PEG-PE loaded with doxorubicin by the pH ammonium sulfate technique is a promising method. It proved to be applicable and successful for treatment of Ehrlich solid tumors implanted in mice and eliminated toxic side effects of doxorubicin.
